# Thiopurines correct the effects of autophagy impairment on intestinal healing – a potential role for ARHGAP18/RhoA

**DOI:** 10.1242/dmm.047233

**Published:** 2021-04-23

**Authors:** Marileen M. C. Prins, Francesca P. Giugliano, Manon van Roest, Stan F. J. van de Graaf, Pim J. Koelink, Manon E. Wildenberg

**Affiliations:** 1 Tytgat Institute for Liver and Intestinal Research, Amsterdam Gastroenterology Endocrinology Metabolism, Amsterdam University Medical Centers (UMC), location AMC, University of Amsterdam, Meibergdreef 9, 1105 AZ Amsterdam, The Netherlands; 2 Department of Gastroenterology and Hepatology, Amsterdam UMC, location AMC, University of Amsterdam, Meibergdreef 9, 1105 AZ Amsterdam, The Netherlands

**Keywords:** SQSTM1/p62, Crohn's disease, 6-TG, Epithelial repair

## Abstract

The ATG16L1 T300A single-nucleotide polymorphism (SNP) is associated with Crohn's disease and causes an autophagy impairment. We have previously shown that this SNP is involved in the migration and hyperactivation of Rac1 in dendritic cells. Mucosal healing, currently the main target for inflammatory bowel disease treatment, depends on restoration of the epithelial barrier and requires appropriate migration of epithelial cells towards and over mucosal lesions. Therefore, we here further investigated the impact of autophagy on epithelial migration.

ATG16L1 knockdown was established in the HT29 human colonic epithelial cell line using lentiviral transduction. Migratory capacity was evaluated using scratch assays and RhoA^GTP^ was measured using G-LISA. Immunofluorescent ARHGAP18 and sequestome 1 (SQSTM1; also known as p62) staining was performed on HT29 cells and primary colonic tissue of Crohn's disease patients.

We observed that ATG16L1 knockdown cells exhibited decreased autophagy and decreased migration capacity. Furthermore, activity of RhoA was decreased. These characteristics were phenocopied using ATG5 knockdown and pharmacological inhibition of autophagy. The migration defect was dependent on accumulation of SQSTM1 and was alleviated upon SQSTM1 knockdown. Strikingly, thiopurines also mitigated the effects of impaired autophagy. RhoA dysregulation appeared mediated through accumulation of the upstream regulator ARHGAP18, which was observed in cell lines, human foetal organoids and primary colonic tissue.

Our results indicate that the ATG16L1 T300A Crohn's disease-associated SNP causes a decrease in migration capacity in epithelial cells, mediated by an increase in SQSTM1 and ARHGAP18 protein and subsequent reduced RhoA activation.

## Abstract (precis)

**Summary:** Analyses of HT29 cells after ATG16L1 knockdown reveal a novel biological mechanism for the association between the autophagy pathway and Crohn's disease, and could aid personalized medicine strategies in Crohn's disease therapy.

## INTRODUCTION

In recent years, genome-wide association studies have been performed and many single-nucleotide polymorphisms (SNPs) have been associated specifically with Crohn's disease. A remarkable proportion of those SNPs is related to autophagy (Barrett et al., 2008; Franke et al., 2010). Autophagy is an intracellular recycling mechanism, degrading misfolded and aggregated proteins, and damaged organelles, as well as balancing sources of energy and eliminating intracellular pathogens (Glick et al., 2010).

Malfunction of the autophagy machinery has been implied in many diseases, including Crohn's disease (Yang and Klionsky, 2020; Jiang and Mizushima, 2014). Genetic variations in genes encoding for autophagy-related 16-like 1 (ATG16L1), Unc-51-like autophagy-activating kinase (ULK1), immunity-related GTPase family M protein (IRGM) and nucleotide-binding oligomerization domain-containing protein 2 (NOD2) have all been associated with Crohn's disease. The ATG16L1 T300A SNP is most prevalent in the general population, with on average 17% homozygosity in healthy controls, ranging from 4% in African and Chinese populations to 23% in Caucasian populations, as found in the International HapMap Project (https://www.genome.gov/10001688/international-hapmap-project). Up to 30% of Crohn's disease patients carry this SNP homozygous, increasing the lifetime risk of developing Crohn's disease approximately twofold (Hampe et al., 2007; Zhang et al., 2009), most strongly in Caucasian carriers. Autophagy is impaired in homozygous carriers, owing to the increased degradation of the ATG16L1 protein, as the T300A SNP creates a cleaving site for caspase-3 (Murthy et al., 2014). The ATG16L1 T300A SNP has been described to affect selective autophagy specifically (Lassen et al., 2014). Homozygous carrying of the T300A SNP has been described to affect intestinal dysbiosis (Sadaghian Sadabad et al., 2015), Paneth cell function (Adolph et al., 2013; Cadwell et al., 2008), regulation of endoplasmic reticulum stress (Adolph et al., 2013) and immune cell function (Wildenberg et al., 2012, 2017).

Cytoskeletal modulation is strongly regulated by RhoGTPases (Etienne-Manneville and Hall, 2002; Kaibuchi et al., 1999). Family members, including RhoA and Rac1, orchestrate the formation and dissolution of actin-rich spots known as focal adhesions, which are crucial for the regulation of cellular movement. Rho activity in the trailing end of the cell is balanced by Rac1 activity on the leading edge (Moorman et al., 1999; Sander et al., 1999). Alterations in the relative activity of these molecules result in imbalance and subsequent inhibition of migration and impaired cellular function (Zhou and Zheng, 2013; Liu et al., 2017; Königs et al., 2014).

We have previously shown that autophagy-deficient dendritic cells exhibit distinct actin cytoskeletal alterations, resulting in decreased migratory capacity. Furthermore, we have also seen that Crohn's disease patients that were homozygous carriers of the ATG16L1 T300A SNP responded better to thiopurine therapy than Crohn's disease patients that carried at least one wild-type (WT) *ATG16L1* allele (Wildenberg et al., 2017). Thiopurines are drugs often used in the treatment of Crohn's disease, but discontinuation of treatment due to side effects is high (Chaparro et al., 2013; Colombel et al., 2010; Qiu et al., 2015).

Although our previous data were obtained in immune cells, autophagy is a process relevant in virtually all cell types, including the intestinal epithelium. The intestinal epithelium has a high restorative capacity, through rapid regeneration of new cells from various subsets of stem cells. However, in order to achieve effective restoration after epithelial injury such as an inflammatory insult, epithelial cells are required to migrate to cover the defect. Dysregulated epithelial migration results in defective restoration of the epithelial barrier. We therefore explored the role of autophagy in the cytoskeletal regulation of cellular migration of intestinal epithelium *in vitro* and *ex vivo*. Using ATG16L1 knockdown constructs *in vitro* that reduced the ATG16L1 protein levels to a similar degree as seen in homozygous ATG16L1 T300A carriers (Murthy et al., 2014), we aimed to elucidate part of the mechanism that makes homozygous carriers prone to developing Crohn's disease. We found that impaired autophagy decreases migration of intestinal epithelial cells after wounding of the monolayer both *in vitro* and *ex vivo*. Interestingly, this phenotype was restored by thiopurines. Furthermore, we found that impaired autophagy affects RhoGTPase homeostasis and results in decreased RhoA activation. This is potentially due to decreased degradation of the RhoGAP ARHGAP18, which was more present in autophagy-impaired intestinal epithelium *in vitro*, *ex vivo* and *in vivo.*

## RESULTS

### Migration capacity is dependent on the autophagy capacity of epithelial cells

**Fig. 1.**
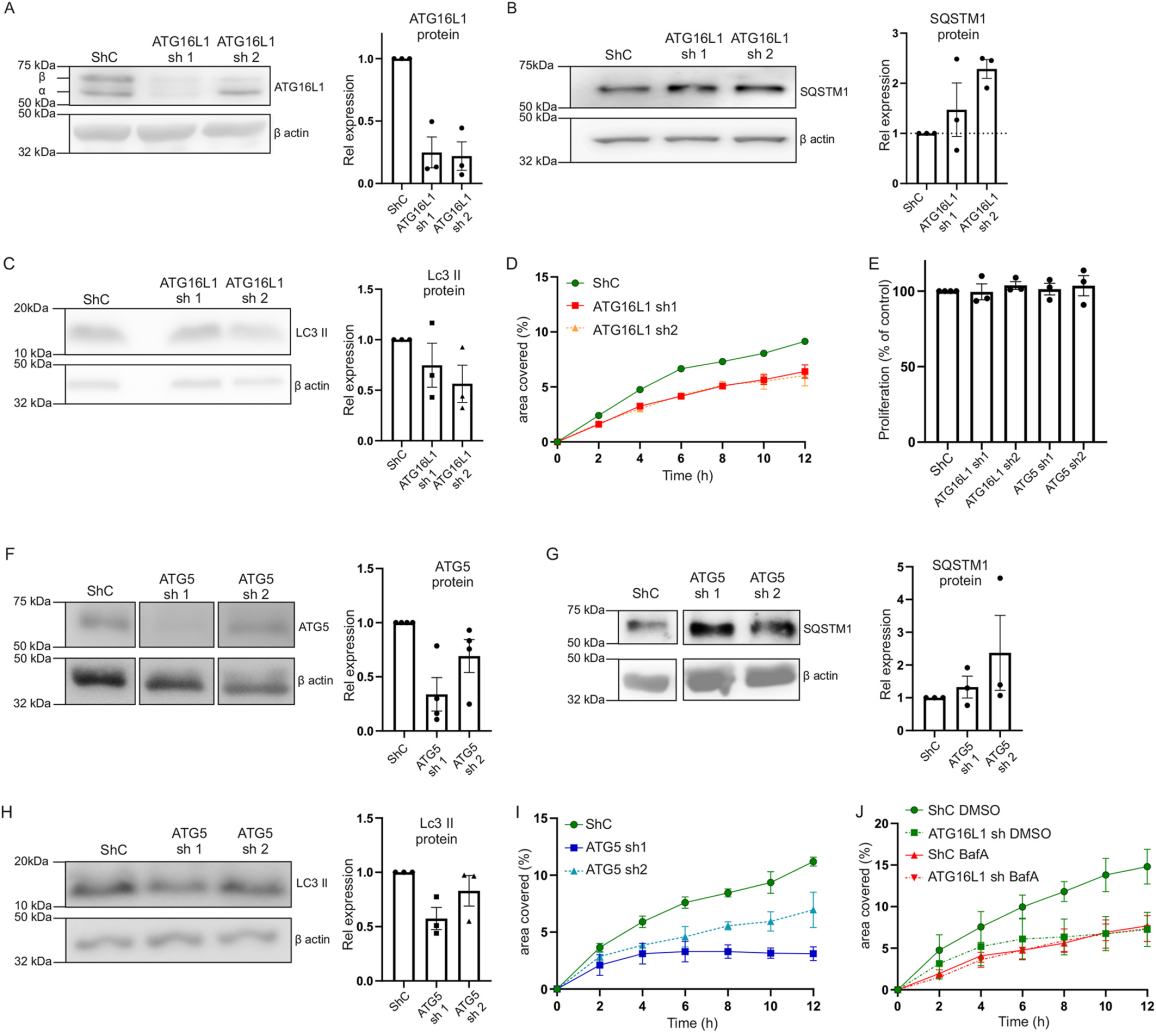
**Autophagy impairment results in decreased migration capacity in HT29 colonic cells.** (A-C) ATG16L1 (A), SQSTM1 (B) and LC3 II (C) protein levels in ATG16L1 knockdown cell lines. (D) Scratch assay in ATG16L1 knockdown cell lines [area under the curve (AUC) *P*<0.0001 for control (ShC, short-hairpin control) versus either *ATG16L1* shRNA construct]. (E) Proliferation measured as EdU incorporation for ATG16L1 and ATG5 knockdown cell lines. (F) ATG5 knockdown; irrelevant lanes removed from single blot. (G,H) SQSTM1 (G) and LC3 II (H) protein levels in ATG5 knockdown cell lines. (I) Scratch assay in ATG5 knockdown cell lines (AUC *P*<0.0001 for control versus either *ATG16L1* shRNA construct). (J) Scratch assay in control and ATG16L1 knockdown cell lines, treated with either DMSO or Bafilomycin A1 (BafA1; 200 nM for duration of the assay) (AUC *P*<0.001 for control DMSO versus sh1 DMSO and control DMSO versus sh1 BafA1; *P*=0.8 for sh1 DMSO and sh1 BafA1). A-D and F-J are representative of *n*=3 individual experiments; E is the combined result of *n*=3 individual experiments. At least two technical replicates were used in D, I and J; three technical replicates were analysed in E. Paired Student's *t*-tests were used in E; D, I and J were analysed using one-way ANOVA using the mean, s.e.m. and *n* based on AUC calculations.

It has previously been shown that the β-isoform of the ATG16L1 protein is rate limiting in the autophagy flux in cells (Murthy et al., 2014). Expression of the β-isoform of the ATG16L1 protein was decreased by lentiviral *ATG16L1* shRNA transduction in HT29 cells, a human colonic epithelial cell line, heterozygous for the ATG16L1 T300A SNP. Knockdown of the β-isoform of the ATG16L1 protein was confirmed at the protein level for two individual ATG16L1 knockdown constructs (Fig. 1A). ATG16L1 knockdown led to impaired autophagy capacity, with increased sequestome-1 (SQSTM1; also known as ubiquitin-binding protein p62) levels (Fig. 1B). As previously reported in ATG16L1 knockout and knockdown cells, LC3 II (also known as MAP1LC3B) levels were slightly decreased in the HT29 ATG16L1 knockdown cell lines (Fig. 1C) (Lassen et al., 2014; Boada-Romero et al., 2016).

Knockdown of ATG16L1 decreased epithelial migration, as measured in a scratch wound healing assay (Fig. 1D; Fig. S1A), whereas proliferation was not affected (Fig. 1E). To determine whether the reduction in migration was a result of the ATG16L1 protein deficiency specifically or an effect of the reduced availability of the ATG5–ATG12–ATG16L1 complex, ATG5 knockdown was established (Fig. 1F). Both ATG5 knockdown cell lines showed impaired autophagy capacity, with increased SQSTM1 levels (Fig. 1G). In accordance with the ATG16L1 knockdown cell lines, LC3 II protein levels were reduced in the ATG5 knockdown cell lines (Fig. 1H). The knockdown of autophagy using these *ATG5* shRNA constructs appeared less than in our ATG16L1 knockdown cell lines. We have previously seen, however, that a reduction in autophagy of merely 30% already had significant impact on Rac1^GTP^ levels in dendritic cells, and we therefore proceeded with the said ATG5 knockdown cell lines (Wildenberg et al., 2017). Furthermore, the ATG5 knockdown cell lines showed reduced migration capacity in the scratch assay (Fig. 1I; Fig. S1B), whereas proliferation again was not affected (Fig. 1E). Finally, to confirm that the reduced migration capacity in ATG16L1 and ATG5 knockdown cell lines was an effect of reduced autophagy capacity, HT29 cell lines were incubated with Bafilomycin A1 (BafA1; 200 nM) during the scratch assay. The migratory capacity of the control cell lines was reduced to the level of the ATG16L1 and ATG5 knockdown cell lines by the chemical inhibition of the autophagic flux (Fig. 1J; Fig. S1C,D), whereas the ATG16L1 and ATG5 knockdown cell lines were hardly affected. This suggests that the migratory capacity in the ATG16L1 and ATG5 knockdown cell lines is impaired by the reduced autophagy capacity and that further reduction of autophagic flux did not have additional effect.

### Autophagy capacity impacts RhoA activity in intestinal epithelium

**Fig. 2.**
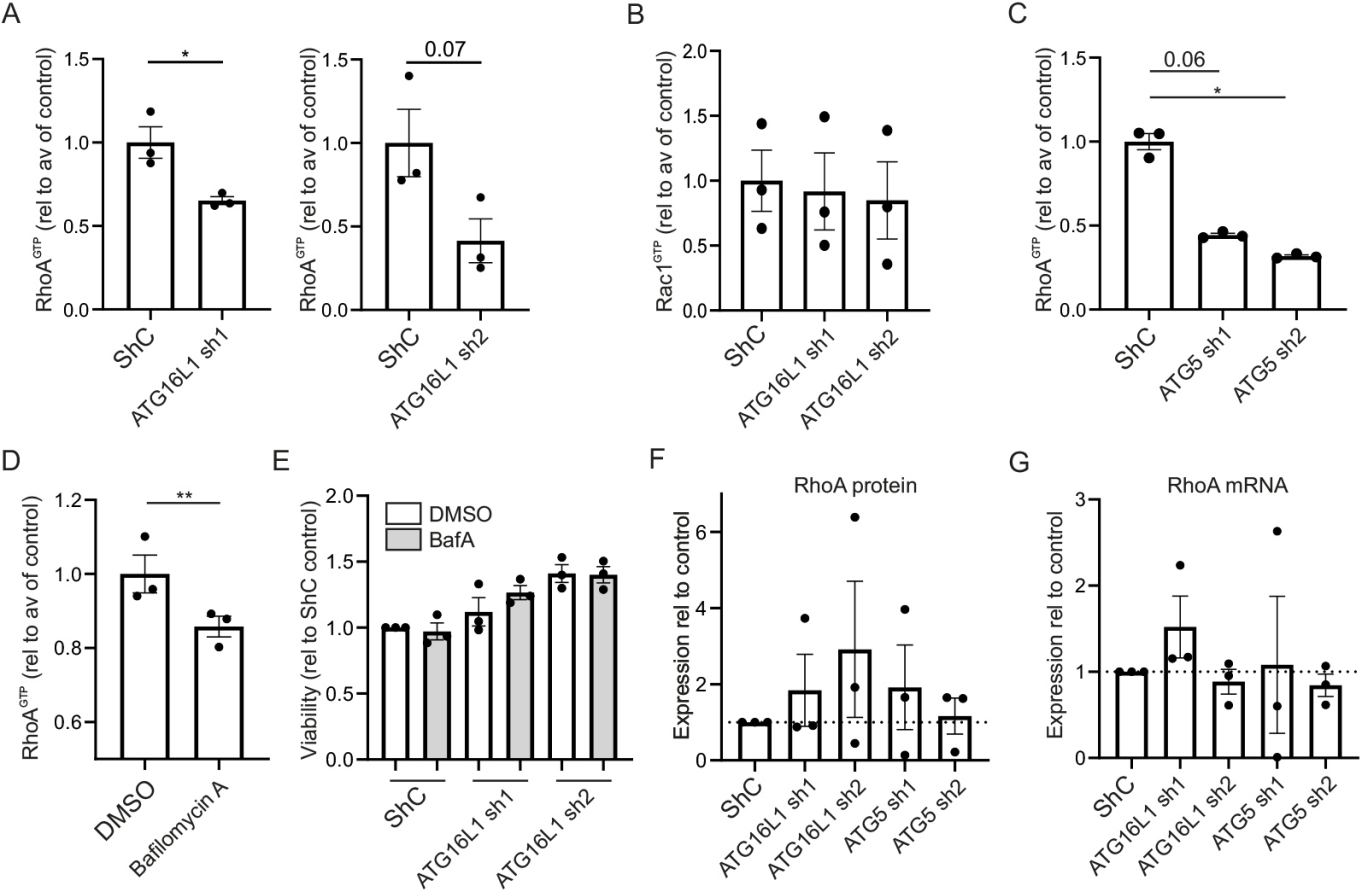
**Autophagy impairment results in decreased RhoA activity in HT29 colonic cells.** (A-D) RhoA^GTP^ (A) and Rac1^GTP^ (B) in ATG16L1 knockdown, RhoA^GTP^ in ATG5 knockdown (C) and the ShC autophagy modulated using BafA1 (200 nM, 2 h; D) in HT29 cell lines as measured by G-LISA. (E) Cytotoxicity of BafA1 (200 nM) was measured by MTS [3-(4,5-dimethylthiazol-2-yl)-5-(3-carboxymethoxyphenyl)-2-(4-sulfophenyl)-2H-tetrazolium] conversion, read after 2 h of incubation in HT29 cell lines. (F) RhoA protein in ATG16L1 knockdown and ATG5 knockdown cell lines, relative to ShC. (G) *RHOA* mRNA expression relative to housekeeping genes cyclovillin and *FSTL1* in HT29 cell lines. Data are representative of at least *n*=3 individual experiments (A,C,D) or combined results of *n*=3 individual experiments (B,E-G). Three technical replicates were analysed in A-D, F and G; six technical replicates were analysed in E. Unpaired Student's *t*-tests were used in A-D; paired Student's *t*-tests were used in E-G. *P<0.05; **P<0.01.

To further investigate the mechanism by which autophagy modulates migratory capacity, we focused on known mediators of the cytoskeleton. RhoGTPases modulate migration via cytoskeletal rearrangement. Active RhoA (RhoA^GTP^) levels were decreased in the ATG16L1 knockdown cell lines (Fig. 2A), whereas Rac1^GTP^ was not altered in either cell line (Fig. 2B).

Similar to the migration phenotype, HT29 ATG5 knockdown phenocopied the ATG16L1 knockdown, with decreased RhoA^GTP^ levels observed as well (Fig. 2C). To confirm that the reduction in RhoA^GTP^ was mediated via the reduction in autophagy, HT29 control cells were incubated with BafA1 for 2 h prior to harvest. Indeed, RhoA^GTP^ was reduced in the autophagy-impaired cells compared to the dimethyl sulfoxide (DMSO) control (Fig. 2D), which was not due to the cytotoxicity of the BafA1 treatment in either cell line (Fig. 2E). A slight trend towards increased total RhoA protein in the ATG16L1 knockdown and ATG5 knockdown cell lines was observed (Fig. 2F). *RHOA* mRNA levels were not altered in both ATG16L1 knockdown and ATG5 knockdown cell lines (Fig. 2G), suggesting regulation on a post-transcriptional or activational level.

### RhoA^GTP^ modulation mimics and rescues the migration phenotype of autophagy deficiency in cells

**Fig. 3.**
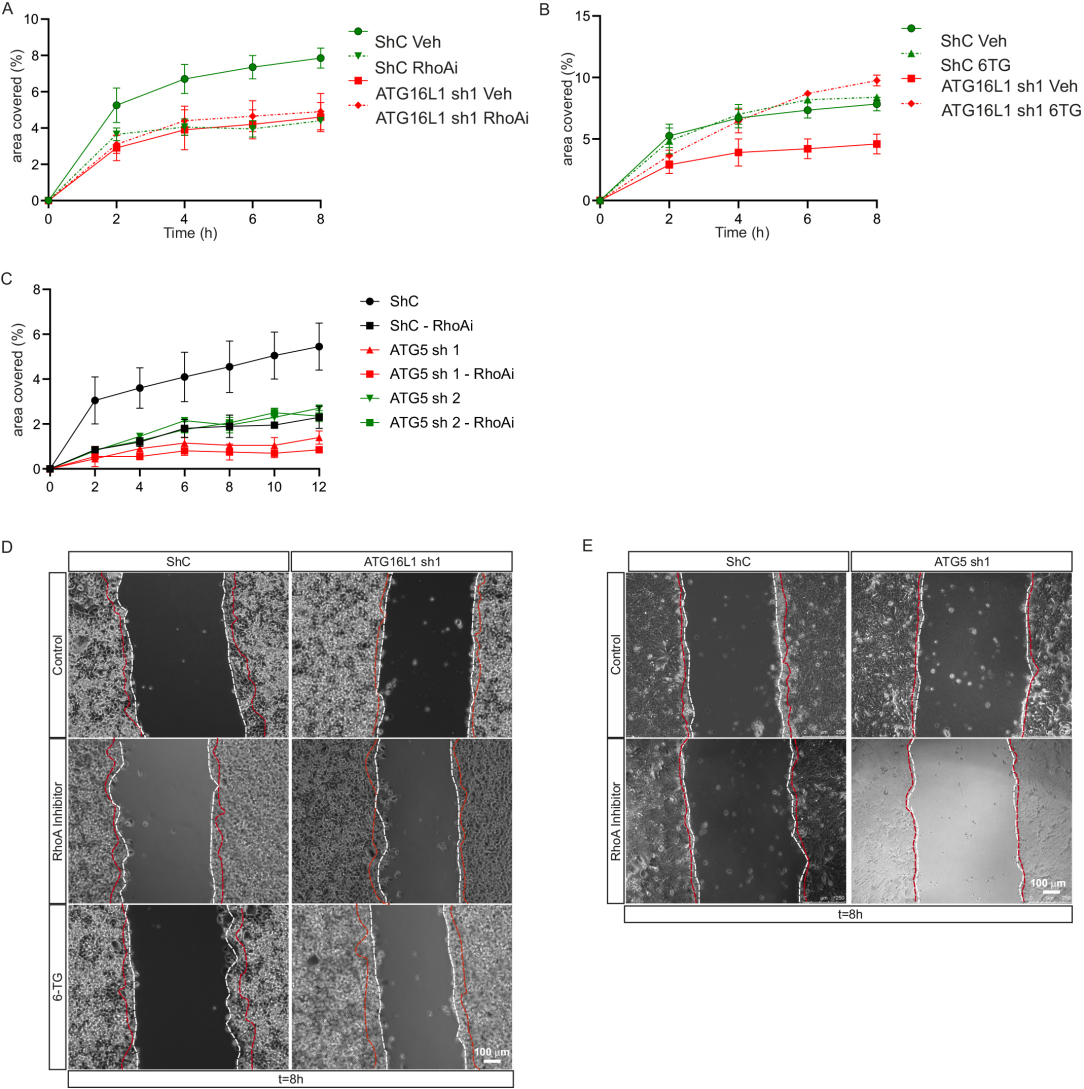
**RhoA activity manipulation mimics and rescues migration capacity in HT29 colonic cells.** (A,B,D) Control and ATG16L1 knockdown cell lines were treated with either vehicle, RhoA inhibitor (RhoAi; RhoA Inhibitor I, Cytoskeleton) (AUC *P*<0.001 for untreated control versus RhoAi, sh1 control and sh1 RhoAi) (A) or 6-TG (AUC *P*=0.9 for untreated control versus 6-TG; *P*=0.0002 for ATG16L1 sh1 control versus 6-TG) (B), depicted at *t*=0 h and *t*=8 h (D). (C,E) Control and ATG5 knockdown cell lines were treated with either vehicle or RhoAi (AUC *P*<0.001 for untreated control versus RhoAi, ATG5 sh1 and sh2; *P*=0.9 for control RhoAi versus untreated ATG5 sh1 and sh2, ATG5 sh1 RhoAi and ATG5 sh2 RhoAi) (C), depicted at *t*=0 h and *t*=8 h (E). In D and E, white dashed lines depict *t*=0 h, red dashed lines depict *t*=8 h. All treatments added for the duration of the experiment. Panels are representative of *n*=3 individual experiments, and at least two technical replicates were analysed per experiment. A-C were analysed using one-way ANOVA using the mean, s.e.m. and *n* based on AUC calculations.

To investigate whether RhoA^GTP^ modulation was key in the migration phenotype observed in autophagy-deficient cells, we aimed to modulate RhoA^GTP^ in the context of autophagy deficiency. The control cell line and ATG16L1 knockdown cell line were incubated with a potent RhoA^GTP^ inhibitor (Rho Inhibitor I, Cytoskeleton, Denver, CO, USA) during the scratch assay. Indeed, migration capacity of the control cells was reduced to the level of the ATG16L1 knockdown cell line (Fig. 3A,D), whereas migration of the ATG16L1 knockdown cell line was hardly affected by the addition of the RhoA inhibitor. Similar results were obtained for the ATG5 knockdown cell lines (Fig. 3C,E).

Interestingly, thiopurines, drugs often used in the treatment of inflammatory bowel disease (IBD), have been shown to prevent activation of Rac1, resulting in a relative shift in the Rac1/RhoA balance towards RhoA (Sander et al., 1999). To evaluate whether thiopurines could correct the phenotype observed in autophagy^low^ and thus RhoA^low^ epithelial cells, the control and ATG16L1 knockdown cell lines were incubated with the thiopurine metabolite 6-thioguanine (6-TG). Indeed, migration capacity of the ATG16L1 knockdown was restored to the level of the control cell line in the HT29 cell line (Fig. 3B,D). In line with an effect dependent on the autophagy-deficient phenotype, the addition of 6-TG to the control cells did not significantly alter migration.

### SQSTM1 knockdown rescues migration phenotype in autophagy-deficient cells

**Fig. 4.**
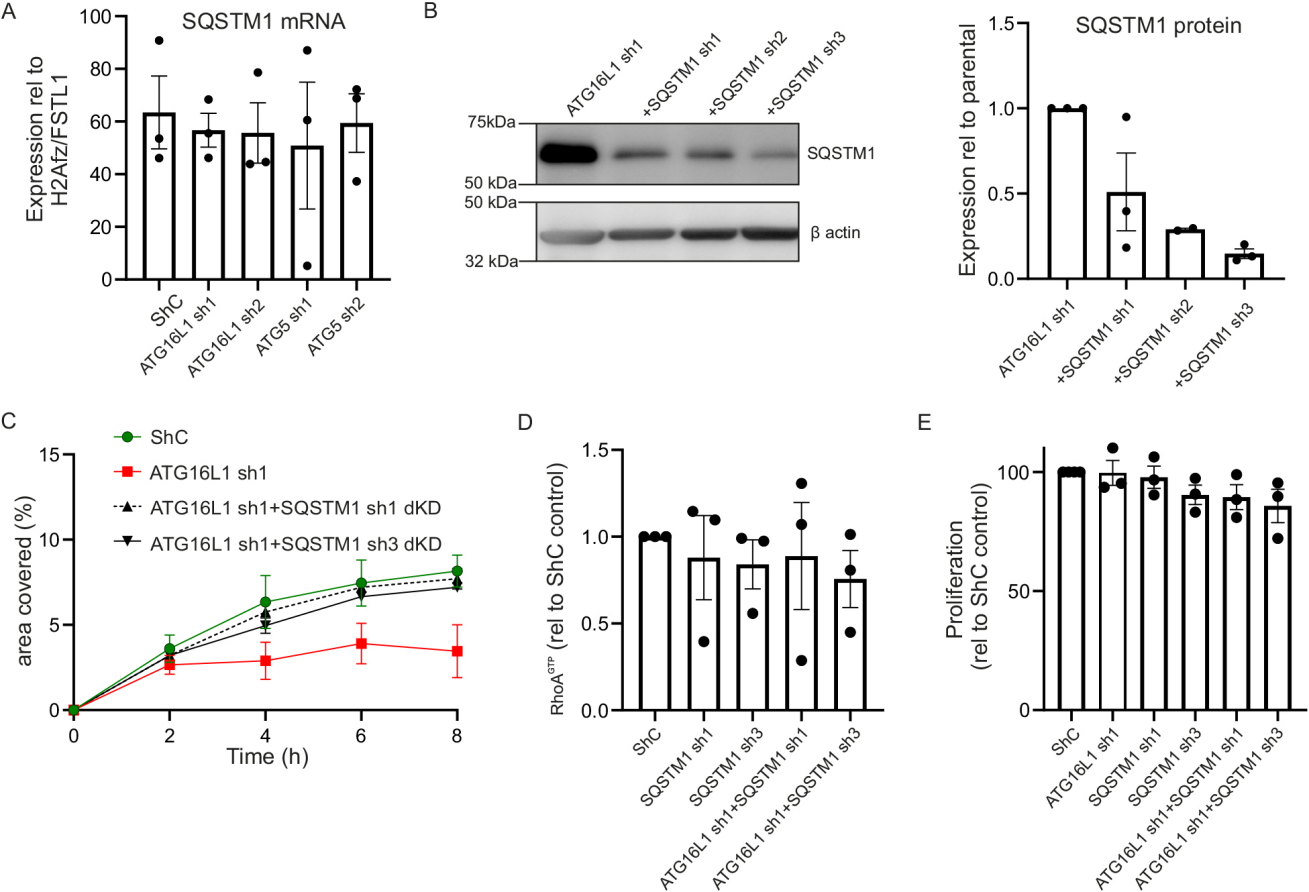
**SQSTM1 knockdown rescues migration capacity in autophagy-impaired HT29 cells.** (A) *SQSTM1* mRNA in control, ATG16L1 knockdown and ATG5 knockdown cell lines. (B) SQSTM1 protein levels in ATG16L1 and ATG16L1+SQSTM1 double-knockdown cell lines. (C) Scratch assay of ATG16L1 and ATG16L1+SQSTM1 double-knockdown cell lines [AUC control versus ATG16L1+SQSTM1 double-knockdown cell lines *P*=0.9 (sh1) and *P*=0.3 (sh3); AUC ATG16L1 sh1 versus ATG16L1+SQSTM1 double-knockdown cell lines *P*<0.0001 (sh1) and *P*=0.008 (sh3)]. (D) RhoA^GTP^ in SQSTM1 knockdown and ATG16L1+SQSTM1 double-knockdown cell lines as measured by G-LISA. (E) Proliferation measured as EdU incorporation for SQSTM1 and SQSTM1+ ATG16L1 knockdown cell lines. Data are combined results (A,D,E) or representative (B,C) of *n*=3 individual experiments. Three technical replicates were analysed in A, D and E; at least two technical replicates were analysed in C. Unpaired Student's *t*-tests were used in A; paired Student's *t*-tests were used in D and E. C was analysed using one-way ANOVA using the mean, s.e.m. and *n* based on AUC calculations.

A decrease in autophagy flux in cells is often characterized by the accumulation of SQSTM1 protein, as also seen in our ATG16L1 and ATG5 knockdown cell lines (Fig. 1B). *SQSTM1* mRNA levels, however, were unchanged (Fig. 4A). We speculated that the accumulation of SQSTM1 interfered with normo-autophagy cell homeostasis and thus RhoGTPase homeostasis. To explore this hypothesis, we lentivirally transduced HT29 control cells and ATG16L1 knockdown cells with independent *SQSTM1* shRNA constructs (Fig. 4B), creating ATG16L1/SQSTM1 double-knockdown cell lines. Two ATG16L1/SQSTM1 double-knockdown cell lines were chosen for further analysis (sh1 and sh3). The migratory capacity of the ATG16L1 double-knockdown cells was completely restored to the level of the control cells, but SQSTM1 knockdown alone did not affect migration in the control cell line (Fig. 4C). Furthermore, RhoA^GTP^ in the ATG16L1 double-knockdown cell lines was restored to the level of the control cells as well (Fig. 4D). Proliferation of the SQSTM1 double-knockdown cell lines was not affected (Fig. 4E) compared to the control cell line. These data suggest that specifically the depletion of SQSTM1 in the autophagy-compromised ATG16L1 knockdown cell line interfered with the effects of autophagy on the cytoskeleton.

### ARHGAP18 is increased in autophagy-deficient cells *in vitro* and *in vivo*

As levels of RhoA protein were not altered strikingly compared to the changes in activity, regulation of the levels of activation appeared likely. Either an increase in a RhoGAP or a decrease in RhoGEF could be underlying the defect in RhoGTPase homeostasis in the context of decreased autophagy. ARHGAP18 is a RhoGAP specific for the conversion of RhoA^GTP^ to RhoA^GDP^ (Maeda et al., 2011).

**Fig. 5.**
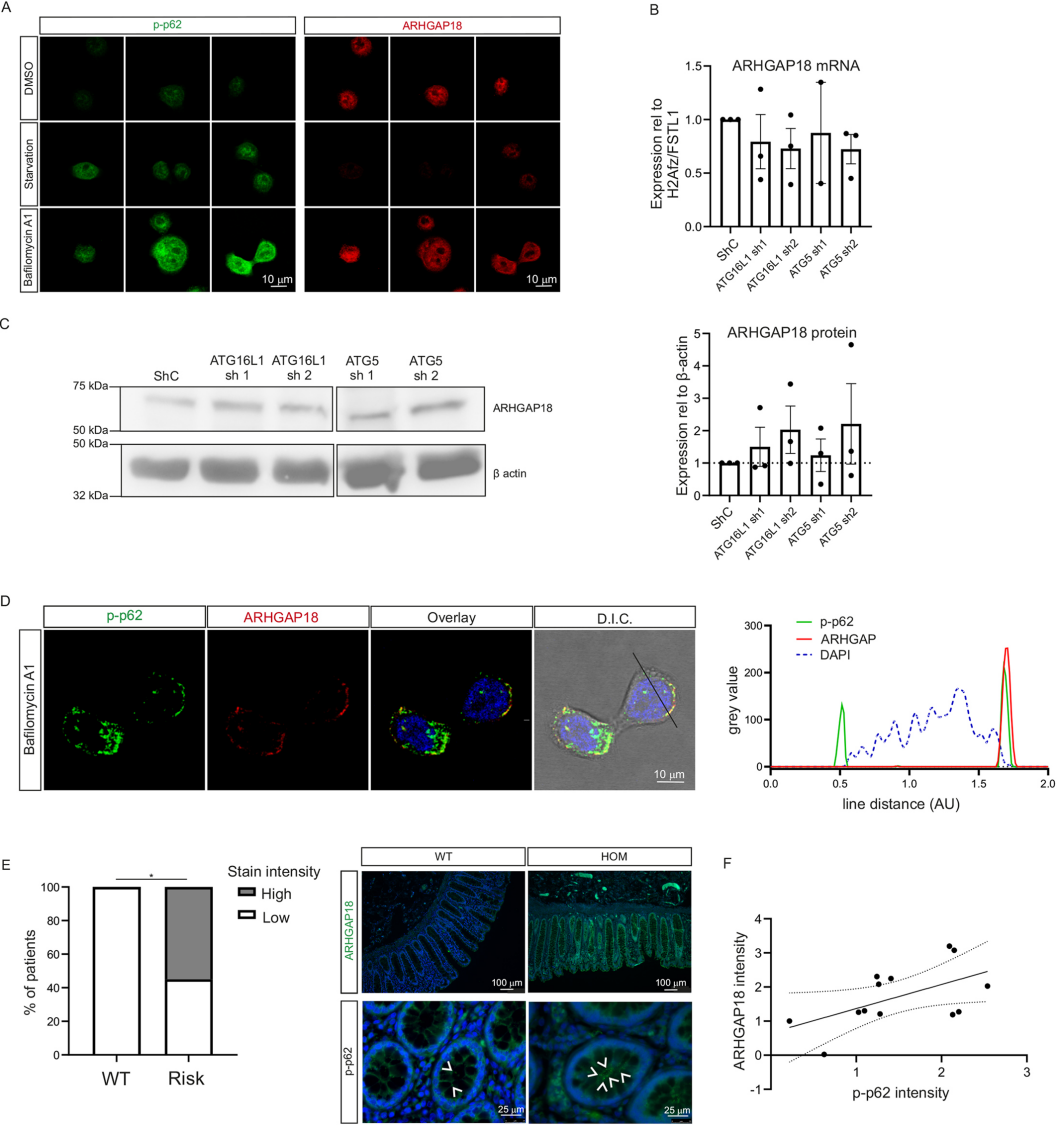
**SQSTM1 and ARHGAP18 correlate with autophagy status *in vitro* and *in vivo*.** (A) HT29 control cells stained for p-SQSTM1 (p-p62; green) and ARHGAP18 (red) after 2 h of DMSO/BafA1 or starvation. (B) *ARHGAP18* mRNA in control, ATG16L1 knockdown and ATG5 knockdown cell lines. (C) ARHGAP18 protein in control, ATG16L1 knockdown and ATG5 knockdown cell lines. (D) Colocalization of p-SQSTM1 (green) and ARHGAP18 (red) in HT29 control cells, treated with BafA1 for 2 h; grey value of cross-section depicted on the right. AU, arbitrary units; D.I.C., differential interference contrast microscopy image. (E) Human intestinal epithelium stained for ARHGAP18 in ATG16L1 WT (*n*=4) and ATG16L1 T300A homozygous carriers (HOM; *n*=9). Representative images are shown in the top row. Bottom row shows p-SQSTM1 staining. Carets indicate clustering of p-SQSTM1. (F) Correlation between ARHGAP18 and p-SQSTM1 staining intensity [*P*=0.04 using chi-square analysis; Spearman *r*=0.5805, 95% CI (0.02511-0.8621)]. At least 30 cells were analysed for A and D; B and C are combined from *n*=3 individual experiments, in which two technical replicates were analysed. Paired Student's *t*-tests were used in B and C; unpaired Student's *t*-tests were used in E.

We found that, upon autophagy modulation using BafA1 for 2 h prior to harvest, ARHGAP18 appeared to slightly increase in the control cell line (Fig. 5A). Similar to *SQSTM1*, the *ARGHAP18* mRNA levels were not changed, implying that the accumulation of ARHGAP18 is independent of transcription (Fig. 5B). The same trend towards increased ARHGAP18 was also found at whole-cell protein level in the autophagy-deficient cell lines with ATG16L1 and ATG5 knockdown (Fig. 5C). Interestingly, whereas both phospho(p)-SQSTM1 and ARHGAP18 appeared increased in an autophagy-impaired condition, ARHGAP18 staining was remarkably reduced upon starvation in an autophagy-competent condition (Fig. 5A).

One possibility is that the increased ARHGAP18 levels are the result of decreased degradation. Cargo destined for autophagic degradation is (poly-)ubiquitinated and targeted to newly forming autophagosomes by adaptor proteins, such as SQSTM1. To see whether ARHGAP18 would be prone to binding to p-SQSTM1, we performed a double staining of p-SQSTM1 and ARHGAP18 in HT29 control cells, treated with DMSO or BafA1 for 2 h prior to fixation. ARHGAP18 did indeed co-locate with p-SQSTM1, in particular on the cell perimeter, where migration is regulated (Fig. 5D).

To conclude, we stained colonic tissue of ATG16L1 WT and homozygous ATG16L1 T300A (HOM) carriers for p-SQSTM1 and ARHGAP18 (Fig. 5E). Tissue was obtained from patients requiring surgery for non-IBD-related disease (*n*=2 WT, *n*=2 HOM) and Crohn's disease patients (*n*=2 WT, *n*=7 HOM). Non-inflamed sections were selected for staining (Fig. S2A). ARHGAP18 and p-SQSTM1 intensity were scored (0-3) while blinded for genotype, based on staining intensity in the epithelial layer. Indeed, an increase in ARHGAP18 protein, scored as grade 2 or higher, was present in the colonic epithelium of ATG16L1 T300A carriers compared to WT carriers. As expected, based on the *in vitro* results, p-SQSTM1 staining intensity correlated with ARHGAP18 intensity in the colonic epithelium [Spearman *r*=0.5805, 95% CI (0.02511-0.8621)] (Fig. 5F).

**Fig. 6.**
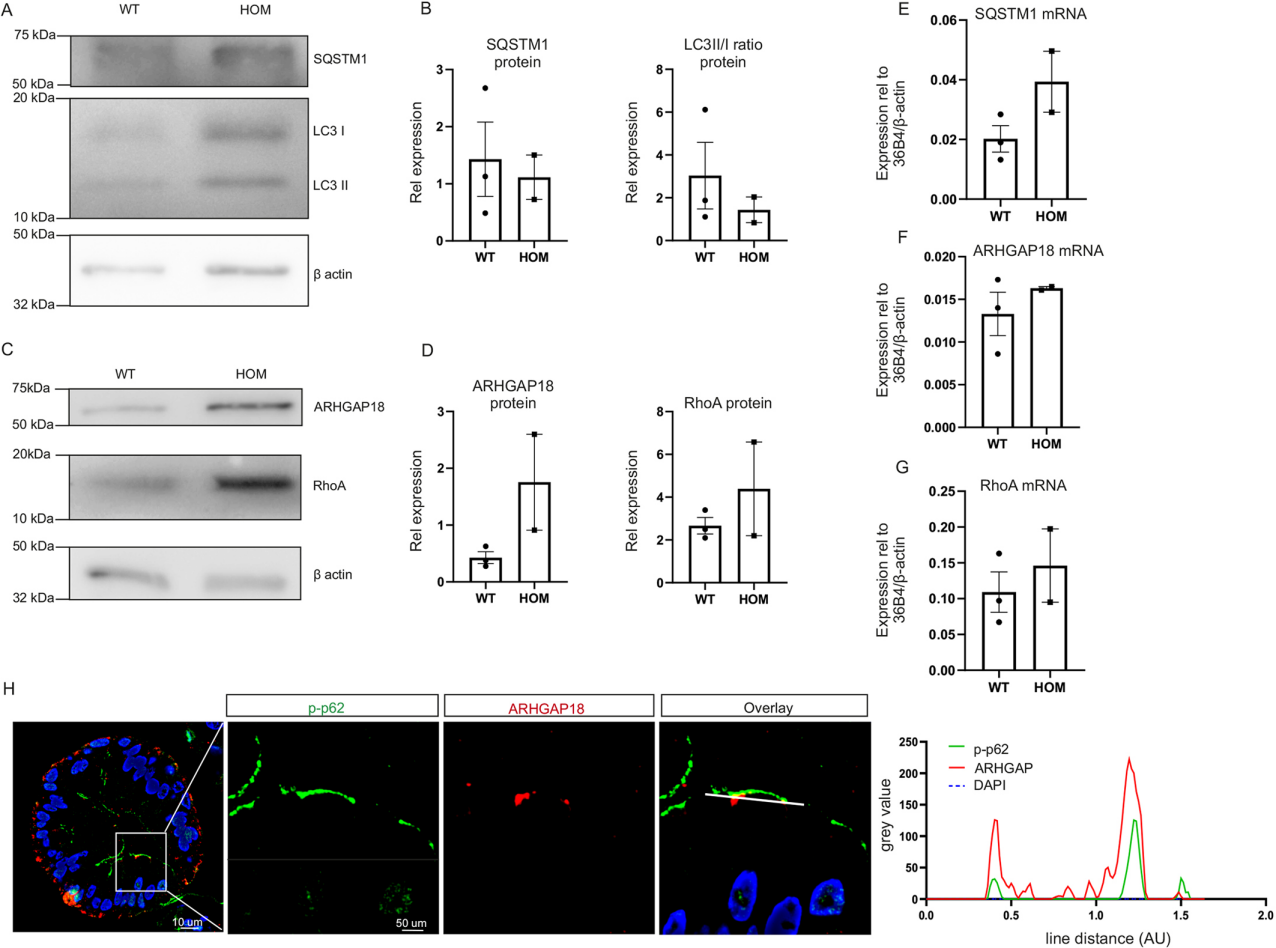
**SQSTM1 and ARHGAP18 correlate with autophagy status in primary human intestinal organoids.** (A-D) LC3 II and SQSTM1 (A,B), and ARHGAP18 and RhoA (C,D) protein levels in WT (*n*=3) and homozygous (*n*=2) human foetal organoids (HFO). (E-G) *SQSTM1* (E), *ARHGAP18* (F) and *RHOA* (G) mRNA in WT (*n*=3) and homozygous (*n*=2) HFO. (H) Homozygous HFO stained for p-SQSTM1 (p-p62) and ARHGAP18; grey value of cross-section depicted on the right.

Thus far, our investigations were performed in the setting of experimental knockdown of ATG16L1 or chemical modulation of autophagy. Finally, to validate the relevance of these findings in a more physiological setting, we isolated human foetal organoids (HFO). Three WT and two homozygous donors were identified. No apparent morphological differences were observed during culture of the HFO (Fig. S2B). As we found in the HT29 ATG16L1 knockdown cell lines, in contrast to our ATG16L1 knockdown cell lines, SQSTM1 levels were not remarkably increased in the homozygous HFO compared to the WT HFO. LC3 II levels, however, did show a similar trend to the HT29 ATG16L1 knockdown cell lines, indicating diminished autophagy in the homozygous HFO (Fig. 6A,B). Furthermore, a trend towards increased ARHGAP18 and RhoA protein levels was observed in the homozygous HFO as was found in the HT29 ATG16L1 knockdown cell lines (Fig. 6C,D). *SQSTM1*, *RHOA* and *ARHGAP18* mRNA levels showed no difference between genotypes (Fig. 6E-G). p-SQSTM1 and ARHGAP18 staining was performed on a homozygous donor (Fig. 6H; Fig. S2C), which showed colocalization as seen in HT29 cells, the increase also particularly concentrated around the cell perimeter.

## DISCUSSION

Crohn's disease is a chronic and disabling form of IBD for many patients. Many SNPs in the autophagy pathway are associated with susceptibility to developing Crohn's disease (Barrett et al., 2008; Franke et al., 2010), although the effective mechanisms remain under investigation. In this paper, we have focused on the patients with Crohn's disease homozygous for the ATG16L1 T300A SNP, accounting for ∼30% of all patients (Hampe et al., 2007). In this subset of patients, selective autophagy is impaired due to the increased degradation of the ATG16L1 protein, as the T300A SNP creates a cleaving site for caspase-3 (Murthy et al., 2014). Using ATG16L1 knockdown constructs that reduced the ATG16L1 protein levels in similar amounts as seen in homozygous ATG16L1 T300A carriers, we evaluated the role of autophagy and its relevant SNP in mechanisms of epithelial repair. We show that, in intestinal epithelium, impaired autophagy alters the activity of RhoGTPases and subsequently impacts proper epithelial migration.

Interestingly, previous studies have linked thiopurines, a class of medications often used in IBD, to regulation of RhoGTPases. The thiopurine metabolite 6-TG destabilizes the Rac1–Vav (also known as VAV1) complex, thus impairing Rac1 activation (Poppe et al., 2006; Tiede et al., 2003). We therefore speculated that, via reducing Rac1^GTP^, 6-TG can indirectly restore the imbalance between Rac1^GTP^–RhoA^GTP^, thereby rescuing/mitigating the migration phenotype of autophagy-deficient epithelial cells. The migration capacity of epithelial cells into damaged intestinal mucosa (wound healing) is crucial for the restoration of the barrier function of the intestinal mucosa (Okamoto and Watanabe, 2016). Despite its wide use, thiopurines are only effective in ∼30% of IBD patients (Colombel et al., 2010). We have previously shown an association between clinical response to thiopurines and the ATG16L1 T300A SNP (Wildenberg et al., 2017). The remarkable restoration of the wound-healing capacity of the HT29 ATG16L1 knockdown cell lines treated with 6-TG may be important in the increased potential of thiopurines in ATG16L1 T300A homozygous Crohn's disease patients compared to ATG16L1 WT Crohn's disease patients.

Side effects reported for thiopurine therapy include gastrointestinal upset, hepatotoxicity and leukopenia, and also an increased risk of developing lymphomas. Approximately one in four patients report side effects during treatment, and ∼15% discontinue treatment because of the side effects (Chaparro et al., 2013; Qiu et al., 2015). As we have entered the age of personalized medicine, and taking into account the high incidence of side effects, selecting Crohn's disease patients based on genotype prior to starting thiopurine therapy might be promising. Genotyping for thiopurine therapy is relatively easy to implement in practice, as has already been done for other genetic variations in thiopurine metabolism in IBD patients (Voskuil et al., 2019), and may select the patient population most likely to benefit from thiopurine therapy.

Here, we show that not the mere decrease in ATG16L1 protein itself, but the effect on the autophagic capacity of the epithelial cells, results in the migration phenotype in HT29 cell lines *in vitro*. Knockdown of ATG5, part of the ATG5–ATG12–ATG16L1 complex, resulted in a similar phenotype to the ATG16L1 knockdown cell lines. Similar results were obtained using the potent autophagy inhibitor BafA1. This is in line with earlier data by Boada-Romero et al. (2016), which show that the T300A SNP alters the ability of the ATG16L1 C-terminal WD40 repeat domain to interact with molecules that recognize this region. Both mechanisms, however, have an effect on the capability of cells to properly execute and initiate autophagy (Murthy et al., 2014; Boada-Romero et al., 2016; Mizushima et al., 2003).

This study shows that RhoA^GTP^ homeostasis is derailed in autophagy-impaired cells. Total RhoA protein was not significantly increased in either ATG16L1 knockdown or ATG5 knockdown cell lines, although it has previously been shown to be targeted for ubiquitination and degradation via autophagy, and an increase in RhoA protein could have led to an increase in its activity as well (Ding et al., 2011). However, the decrease in RhoA^GTP^ of ∼30% is reminiscent of the increase in Rac1^GTP^ of ∼30% that our group has previously found in autophagy-impaired dendritic cells (Wildenberg et al., 2017), although we did not observe increased Rac1 activity in epithelial cells. One explanation could be the different importance of either RhoGTPase in different cell types, as well as different spatiotemporal modes of action of these two RhoGTPases in different cell types (Wang and Zheng, 2007). Second, overexpression of ARHGAP18 has been shown to regulate RhoA, but not Rac1 and CDC42 activity (Maeda et al., 2011). By inhibiting RhoA^GTP^ chemically, which resulted in a similar effect on the migratory capacity as autophagy impairment, we provide a link between impairment of autophagy and impairment of migration. Although we have not been able to determine RhoA activity in the HFO, as cell lysis for G-LISA and/or a pulldown assay on western blot requires disruption of the organoid structure and thereby may potentially affect RhoGTPase activities substantially, such experiment would be most informative in future research into RhoGTPase homeostasis.

RhoGTPases are modulated via GTPase activating proteins (GAPs) and guanine nucleotide exchange factors (GEFs) (Etienne-Manneville and Hall, 2002). A reduction in RhoA^GTP^ is potentially due to an accumulation of a RhoGAP or a decrease in a RhoGEF. We have focused on ARHGAP18 as this RhoGAP is one of few RhoGAPs relatively specific for the activation of RhoA (Maeda et al., 2011). The accumulation of ARHGAP18 protein in autophagy-impaired cells was evident in HT29 cell lines and in colonic tissue of homozygous ATG16L1 T300A carriers. However, we cannot exclude other RhoGAPs or RhoGEFs contributing to altered RhoA^GTP^ in autophagy-impaired epithelial cells.

The accumulation of ARHGAP18 in autophagy-impaired cells could be the result of reduced degradation via autophagy itself, as we have shown that ARHGAP18 is prone to autophagic degradation upon starvation. It could also be that ARHGAP18 protein accumulates in autophagy-deficient cells due to binding to accumulated SQSTM1. As SQSTM1 accumulates in the cell, it is phosphorylated at S407 and S403 (p-SQSTM1). p-SQSTM1 has increased binding capacity for any (poly-)ubiquitinated protein as well as other p-SQSTM1 proteins, forming large clusters known as sequestomes (Matsumoto et al., 2011). Abundance of these sequestomes has been associated with various disease states, including neurodegenerative diseases, tumorigenesis, cardiometabolic diseases, myopathies and liver disease (Katsuragi et al., 2015). Concomitant knockdown of SQSTM1 in the HT29 autophagy-deficient intestinal epithelial cells completely rescued the migration phenotype, suggesting that SQSTM1 has a pivotal role in the homeostasis of RhoGTPase activity in epithelial cells. Microscopic analysis indeed showed colocalization of ARHGAP18 and p-SQSTM1 in epithelial cells and HFO, in particular at the migrating edges, supporting this hypothesis. However, whether this is due to the binding of SQSTM1 to ubiquitinated proteins and their accumulation in sequestomes is solely responsible for the phenotype observed or other known signalling functions of SQSTM1 also play a role (Katsuragi et al., 2015; Ma et al., 2019) remains to be elucidated.

In conclusion, we have shown that, in epithelium, autophagy deficiency derails RhoGTPase homeostasis. The reduced RhoA^GTP^ causes defective migration, which can be corrected with 6-TG/thiopurine treatment as well as reduction of SQSTM1 in the cell. Binding of SQSTM1 and p-SQSTM1 to the RhoGAP ARHGAP18 may be the underlying mechanism that connects autophagy impairment and RhoA^GTP^ homeostasis. These mechanisms support the observation that Crohn's disease patients homozygous for the ATG16L1 T300A SNP are more likely to benefit from thiopurine therapy, opening the future towards personalized medicine in this patient group.

## MATERIALS AND METHODS

### Cell culture

HT29 (ACC299, DSMZ, Braunschweig, Germany) cells were seeded at 3-4×10^5^ cell/cm^2^ cell density. Both were refreshed twice weekly in Dulbecco's modified Eagle medium (DMEM; Lonza, Leusden, The Netherlands), 10% foetal calf serum (FCS), penicillin/streptomycin (Invitrogen, Thermo Fisher Scientific, Waltham, MA, USA) and 5 mM glutamine (Lonza). Prior to cell harvest for protein analysis or fixation for immunofluorescent staining, HT29 cells were starved in PBS for 2 h to induce an autophagy response.

### HFO culture

HFO were isolated from human foetal intestinal tissues (gestational age 18-20 weeks), which were received with the approval of the Amsterdam UMC ethical committee. Tissues were obtained from the Bloemenhove clinic (Heemstede, The Netherlands) by the HIS Mouse Facility of the Amsterdam UMC. A written informed consent for the use of tissue for research purposes was signed by each donor. The experimental procedures were approved by the HIS Mouse Facility (Amsterdam UMC) and performed according to the Amsterdam UMC Research Code-relevant guidelines and regulations, in line with the Declaration of Helsinki.

The isolation of the intestinal stem cells (ISCs) was performed according to a previous protocol (Sato et al., 2011, 2009). In short, the tissue was macroscopically divided into proximal and distal small intestine, cut open longitudinally, gently scraped with a coverslip, washed in cold PBS and then incubated for 30 min with 2 mmol/l EDTA in PBS at 4°C on a roller bank. Subsequently, dissociated epithelial cells were collected by washing extensively with 10% FCS in PBS, and then passed through a cell strainer (70 µm) and precipitated by centrifugation at 110 ***g*** for 5 min at 4°C. The pellet obtained was resuspended in 10 μl Matrigel (Corning). In order to allow the Matrigel to solidify, the plate was incubated for 10-15 min at 37°C, after which 0.5 ml human intestinal stem cell medium (HISC) was added to each well. HISC contained Advanced DMEM/F12 supplemented with 1× GlutaMAX, 0.01 M Hepes, 0.2 U/ml penicillin/streptomycin, 1× B27 supplement, 1× N2 supplement, 0.05 μg/ml mouse epidermal growth factor (EGF) (all Invitrogen), 1.25 mM n-acetyl-L-cysteine, 10 nM [Leu15]-Gastrin, 10 mM nicotinamide, 10 μM SB202190 (all Sigma-Aldrich), 500 nM A83-01 (Tocris), 20% mNoggin, 10% R-spondin, 50% WNT3A (all three Amsterdam UMC conditioned home-made media). The medium was refreshed every 3-4 days. Every 5-7 days, organoids were passaged by mechanical disruption as described previously (Sato et al., 2011, 2009).

### Genotyping

DNA was isolated and precipitated from each cell line, HFO donor and colonic tissue donor. Cells were lysed in lysis buffer (100 mM Tris-HCl pH 8.5, 5 mM Na_2_EDTA, 200 mM NaCl, 0.2% SDS) with Protease K (100 µg/ml), incubated at 56°C for 30 min and 85°C for 15 min. Restriction fragment length polymorphism (RFLP) was used to genotype for the autophagy-related SNP ATG16L1 T300A (rs2241880) (Ota et al., 2007).

For primer sequences and RFLP details, see Table 1. Fragments were separated using gel electrophoresis.

**Table 1.**
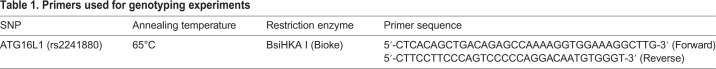
**Primers used for genotyping experiments**

### Quantitative RT-PCR analysis

mRNA was isolated using the Bioline ISOLATE II RNA Mini kit (BIO-25073, Bioline, London, UK) according to the manufacturer's protocol. cDNA was generated using RevertAid reverse transcriptase (Thermo Fisher Scientific, Landsmeer, The Netherlands) and random primers (Promega, Leiden, The Netherlands). Quantitative RT-PCR was performed on a Bio-Rad iCycler using a SensiFAST SYBR No-ROX Kit (GC Biotech Bio-98020, Waddinxveen, The Netherlands) according to the manufacturer's protocol. Intron-spanning primers were designed for RhoA, ARHGAP18 and SQSTM1; melting curves and product size were used to validate specificity. Sequences can be found in Table 2. For relative expression, all data were normalized against expression of the stable reference genes cyclophilin and *FSTL1*, as identified by GeNorm algorithm (Vandesompele et al., 2002). Relative expression levels were calculated using LinRegPCR software (version 2015.4, Amsterdam, The Netherlands).

**Table 2.**
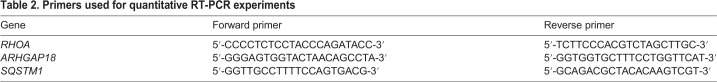
**Primers used for quantitative RT-PCR experiments**

### Lentivirus transduction

The *ATG16L1* TRCN0000137486 and TRCN0000138556, *ATG5* TRCN0000150940 and TRCN0000151474 and/or *SQSTM1* TRCN0000007233 and TRCN0000007235 (all Sigma-Aldrich, Zwijndrecht, The Netherlands) shRNA constructs were used for knockdown of ATG16L1, ATG5 and/or SQSTM1 in HT29 cells. A non-targeting shRNA (SHC002, Sigma-Aldrich) was used as a control for the knockdown cell lines. Transduction was performed using DEAE Dextran (Amersham Pharmacia Biotech, Piscataway, NJ, USA), and selection was performed using 10 µg/ml puromycin (Sigma-Aldrich) for at least 7 days continuously and weekly addition of puromycin to the medium after initial selection. (Stable) knockdown of relevant genes was routinely checked by western blot analysis. See Table 3 for sequences of all used lentiviral inserts.

**Table 3.**
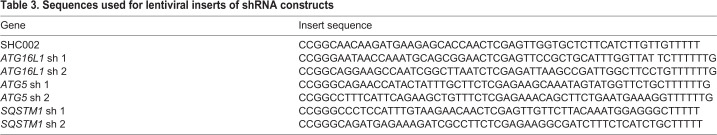
**Sequences used for lentiviral inserts of shRNA constructs**

### Protein analysis

For western blot analysis, cells were lysed in Cell Lysis Buffer (Cell Signaling Technology, Leiden, The Netherlands) or RIPA buffer (0.15 M NaCl, 0.05 M Tris-HCl pH 7.5, 1% NP40, 0.5% DCA, 0.1% SDS, 0.1 mM EDTA) and boiled in sample buffer containing 0.25 M Tris-HCl pH 6.8, 8% SDS, 30% glycerol, 0.02% Bromophenol Blue and 3% b-mercaptoethanol. Samples were run on 10%, 15% and 10-18% gradient SDS-PAGE gels under reducing conditions and transferred to an Immobilon-P PVDF membrane (Millipore, Burlington, MA, USA). Membranes were blocked by incubation in 5% bovine serum albumin (BSA) or 5% milk powder (Protifar Plus, Alliance Healthcare Nederland) in TBS-T (Tris-buffered saline with 0.1% Tween^®^ 20 detergent). Antibodies used for detection were as follows: anti-β-actin (Sigma-Aldrich; 1:10,000), anti-SQSTM1 (clone 3/P62, 610833, BD Biosciences, San Jose, CA, USA; 1:2000), anti-RhoA (clone 1B12, ab54835, Abcam, Cambridge, UK; 1:1000), anti-ARHGAP18 (ab106553, lot GR56014-12, Abcam; 1:1000), anti-LC3a/b (LC3 A/B, clone D3U4C, Cell Signaling Technology; 1:1000), anti-ATG16L1 (clone D6D5, 8089S, Cell Signaling Technology; 1:1000) and anti-ATG5 (clone D5F5U, 12994T, Cell Signaling Technology; 1:1000). Horseradish peroxidase (HRP)-conjugated secondary antibodies were used accordingly [anti-mouse goat IgG HRP conjugated (GAMPO), P044701 and anti-rabbit goat IgG HRP conjugated (GARPO), P044801; both DAKO, Santa Clara, CA, USA; 1:2000]. Expression was detected by Lumilight Plus (Roche, Woerden, The Netherlands).

### Proliferation assay

Cell proliferation was measured using a Click-iT^®^ plus EdU Alexa Fluor^®^ 647 Flow Cytometry Assay Kit (Invitrogen, Thermo Fisher Scientific). Cells were seeded 50,000 per well in a 12-well plate and grown for 24 h prior to analysis in full culture medium without reaching confluence. After 24 h, 5-ethynyl-2′-deoxyuridine (EdU) was added for 2 h. All flow cytometry results were obtained using a FACS Fortessa (BD Biosciences) and analysed using FlowJo software version 10.3 (Treestar, Ashland, OR, USA).

### Cytotoxicity assay

Cytotoxicity of BafA1 (B1793-10UG, Sigma-Aldrich) in HT29 cell lines was measured using a CellTiter 96^®^ AQueous One Solution Cell Proliferation Assay System (Promega). Cells were cultured 24 h prior to analysis without reaching confluence and treated with DMSO, BafA1 (200 nM) or 6-TG (10 µM, 6-Thioguanine A4882, Sigma-Aldrich) for 2 h. Results were measured every 30 min at 490 nm for 2.5 h.

### Scratch assay

Cells of the HT29 cell lines were seeded at high density for 12-24 h in full culture medium prior to assay in a 12-well format, reaching confluence at *t*=0. Wounding of the confluent monolayer was performed by scratching the surface with a 200 µl pipette tip. Directly after scratching the monolayer, 6-TG (10 µM), RhoA inhibitor (50 nM, RhoA Inhibitor I, Cytoskeleton, Denver, CO, USA), BafA1 (200 nM) or DMSO were added, and microscopic imaging of the scratch was started.

All cell migration was filmed overnight with a DMi8 inverted microscope (Leica, Wetzlar, Germany), fitted with a humidified culture chamber maintained at 37°C. Analysis was performed using ImageJ software (http://imagej.nih.gov/ij/).

### RhoA^GTP^ and Rac1^GTP^ assay

The phosphorylated, active GTP-bound conformational state of RhoA protein, RhoA^GTP^, was measured using RhoA G-LISA Activation Assay (Luminescence format, Cytoskeleton). The phosphorylated, active GTP-bound conformational state of Rac1 protein, Rac1^GTP^, was measured using Rac1 G-LISA Activation Assay (Luminescence format, Cytoskeleton). Cells were cultured 24 h prior to harvest in full culture medium. Cell lysis, protein concentration and assay were performed according to the manufacturer's manual.

### Immunofluorescent staining

Cells were cultured onto glass coverslips for 24 h prior to fixating for the colocalization studies. Cells were fixed using 4% formaldehyde solution (VWR, Amsterdam, The Netherlands) and permeabilized in 0.05% Triton X-100 in PBS (Bio-Rad, Veenendaal, The Netherlands). Slides were blocked with 0.5% BSA in PBS and incubated overnight at 4°C with anti-ARHGAP18 (ab175970, Abcam; 1:100) and anti-p-SQSTM1 (Ser403, D344-3MS, MBL; 1:500). The slides were stained at room temperature with fluorescent-labelled secondary antibody (donkey-anti-rat AF488, A21208, lot 1476598, Life Technologies; donkey-anti-rabbit AF546, A10040, lot 1833519, Invitrogen; 1:200). Slides were mounted with ProLong Gold Antifade reagent with 4′,6-diamidino-2-phenylindole (DAPI) (Thermo Fisher Scientific). Images were taken with a Leica DM6000 microscope using a 63× objective and 10× ocular, and LAS AF software (Leica).

### Immunohistochemistry

Fresh tissue was fixed in 4% formaldehyde solution (VWR) at room temperature, and embedded in paraffin and sectioned at 4 µm. HFO were fixed in 4% formaldehyde solution (VWR) for 1 h at 4°C and embedded in HistoGel (Thermo Fisher Scientific) prior to paraffin embedding and sectioned at 4 µm. Paraffin slides were deparaffinized and rehydrated. For antigen retrieval, slides were treated at 96°C for 10 min in 0.01 M sodium citrate buffer pH 6.0. Sections were blocked with PBT (PBS, 0.1% Triton X-100, 1% w/v BSA) and incubated overnight at 4°C with primary antibody anti-ARHGAP18 (ab175970, Abcam; 1:100) or anti-p-SQSTM1 (5 µg/ml; MBL). Tissue sections were stained at room temperature with fluorescent-labelled secondary antibody (goat anti-rabbit Alexa Fluor 488, A11008, lot 1966932, and goat anti-rat Alexa Fluor 546, A11081, lot 1661229; both Invitrogen; 1:500). Slides were mounted with ProLong Gold Antifade reagent with DAPI (Thermo Fisher Scientific). Images were taken with a Leica DM6000 microscope, using a 10× objective and 7× ocular and LAS AF software. Scoring of the immunohistochemistry slides was performed blinded for genotype. Specimen collection of primary colonic tissue was approved by the biobank review committee of the Academic Medical Center Amsterdam (number 178#A201470).

### Statistics

Statistical analysis was performed with Prism 8.0.2 software (GraphPad, La Jolla, CA, USA). Graphs depict mean±s.e.m.; paired or unpaired Student's *t*-test and chi-square test were used where appropriate, as indicated in figure legends. *P*<0.05 was considered significant.
